# Supplementary material for: Heat adaptation of phage T7 under an extended genetic code
Source: Virus Evol. 2021 Dec 1;7(2):veab100. doi: 10.1093/ve/veab100 (PMC8923235; doi:10.1093/ve/veab100)
Supplement: veab100_Supp [file veab100_supp.zip › Sequencing_Analysis.zip!/Sequencing_Analysis/A3/evidence/SNP_13.html]

BRESEQ :: Evidence


| Predicted mutation | | | | | | |
| --- | --- | --- | --- | --- | --- | --- |
| evidence | seq id | position | mutation | annotation | gene | description |
| RA | NC\_001604.1 | 29,258 | A→G | intergenic (–/–) | – / – | –/– |

| Read alignment evidence... | | | | | | | | | | | |
| --- | --- | --- | --- | --- | --- | --- | --- | --- | --- | --- | --- |
|  | seq id | position | | ref | new | freq | score (cons/poly) | reads | annotation | genes | product |
| \* | NC\_001604.1 | 29,258 | 0 | A | G | 99.0% | 4626.8 / NA | 1534 | intergenic (–/–) | *–/–* | –/– |
| Reads supporting (aligned to +/- strand):  **ref** base A (2/2);  **major** base G (754/759);  **minor** base T (10/5);  **total** (767/767) | | | | | | | | | | | |
| Fisher's exact test for biased strand distribution *p*-value = 2.99e-01 | | | | | | | | | | | |
| Kolmogorov-Smirnov test that lower quality scores support variant *p*-value = 1.00e+00 | | | | | | | | | | | |

Only 100 of 1535 total aligned reads displayed.  

`GCGTCGTCCAGACTCTGCTGACTTCTTTGAGAAGTATATCGACAACGGTCTGGTTACTGGCGCAATCCCATCTGATGCTCAAGCCACACAGCTTATAAGCCAAGCGTTCAGTGACGCTTCTAGCCGTGCTGGTGGTGCTGACTTCCTGATGCGAGTCGGTGACAAGAAGGTAACACTTAACGGAGCCACTACGACTTACCGAGAGTTGATTGGTGAGGAACAGTGGAACGCTCTCATGGTCACAGCACAACGTTCTCAGTTTGAGACTGACGCGAAGCTGAACGAGCAGTATCGCTTGAAGATTAACTCTGCGCTGAACCAAGAGGACCCAAGGACAGCTTGGGAGATGCTTCAAGGTATCAAGGCTGAACTAGATAAGGTCCAACCTGATGAGCAGATGACACCACAACGTGAGTGGCTAATCTCCGCACAGGAACAAGTTCAGAATCAGATGAACGCATGGACGAAAGCTCAGGCCAAGGCTCTGGACGATTCCATGAAGTCAATGAACAAACTTGACGTAATCGACAAGCAATTCCAGAAGCGAATCAACGGTGAGTGGGTCTCAACGGATTTTAAGGATATGCCAGTCAAC  >  NC_001604.1/28960‑29554`  

                                                                                                                                                                                                                                                                                                          `|`                                                                                                                                                                                                                                                                                                          
  `gtcgtcCAGACTCTGCTGACTTCTTAGAGAAGTATATCGACAACGGTCTGGTTACTGGCGCAATCCCATCTGATGCTCAAGCCACACAGCTTATAAGCCAAGCGTTCAGTGACGCTTCTAGCCGTGCTGGTGGTGCTGACTTCCTGATGCGAGTCGGTGACAAGAAGGTAACACTTAACGGAGCCACTACGACTTACCGAGAGTTGATTGGTGAGGAACAGTGGAACGCTCTCATGGTCACAGCACAACGTTCTCAGTTTGAGACTGACGCGAAGCTGAACGAGCAGTATCGCTTGGAGAt                                                                                                                                                                                                                                                                                                      <  1:222386/301‑1 (MQ=255)`  
     `gtcCAGACTCTGCTGACTTCTTTGAGAAGTATATCGACAACGGTCTGGTTACTGGCGCAATCCCATCTGATGCTCAAGCCACACAGCTTATAAGCCAAGCGTTCAGTGACGCTTCTCGCCGTGCTGGTGGTGCTGACTTCCTGATGCGAATCGGTGACAAGAAGGTAACACTTAACGGCGCCACTACGACTTACCGAGAGTTGATTGGTGAGGAACAGTGGAACGCTCTCATGGTCACAGCACAACGTTCTCAGTCTGAAACTGACGCGAAGCTGAACGAGCAGTATCGCTTGGAGATTaa                                                                                                                                                                                                                                                                                                   >  1:70087/1‑301 (MQ=255)`  
      `tcCAGACTCTGCTTACTTCTTTGAGAAGTATATCGACAACGGTCTGGTTACTGGCGCAATCCCATCTGATGCTCAAGCCACACAGCTTATAAGCCAAGCGTTCAGTGACGCTTCTAGCCGTGCTGGTGGTGCTGACTTCCTGATGCGAGTCGGTGACAAGAAGGTAACACTTAATGGAGCCACTACGACTTACCGAGAGTTGATTGGTGAGGAACAGTGGAACGCTCTCATGGTCACAGCACAACGTTCTCAGTTTGAGACTGACGCTAAGCTGAACGAGCAGTATCGCTTGGAGATTAAc                                                                                                                                                                                                                                                                                                  <  1:143181/301‑1 (MQ=255)`  
      `tcCAGACTCTGCTTACTTCTTTGAGAAGAAAATCGCAAACGGTCGGGTTACTGGCGCAATCCCATCTGATGCTCAAGCCACACAGCTTATAAGCCAAGCGTTCAGTGACGCTTCTAGCCGTGCTGGTGGTGCTGACTTCCTTATGCGAGTCGGTGACAAGAAGGTAACACTTAACGGAGCCACTACGACTTACCGAGAGTTGATTGGTGAGGAACAGTGGAACGCTCTCATGGTCACAGCACAACGTTCTCAGTTTGAGACTGACGCGAAGCTGAACGAGCAGTATCGCTTGGAGATTAAc                                                                                                                                                                                                                                                                                                  <  1:190436/301‑1 (MQ=255)`  
      `tcCAGACTCTGATGACTTCTTTGAGAAGTATATCGACAACGGTCTGGTTACTGGCGCAATCCCATCTGATGCTCAAGCCACACAGCTTATAAGCCAAGCGTTCAGTGACGCTTCTAGCCGTGCTGGTGGTGCTGACTTCCTGATGCGAGTCGGTGACAAGAAGGTAACACTTAACGGAGCCACTACGACTTACCGAGAGTTGATTGGTGAGGAACAGTGGAACGCTCTCATGGTCACAGCACAACGTTCTCAGTTTGAGACTGACGCGAAGCTGAACGAGCAGTATCGCTTGGAGATTAAc                                                                                                                                                                                                                                                                                                  <  1:236164/301‑1 (MQ=255)`  
        `cAGACCTTGCTTACTTCTTTGGTAAGTTTATCGGAAACGGTCTTGGTACTTGGGCAAACCCATCTGATGCTCAAGCCACACAGCTTATAAGCCAAGCGTTCAGTGACGCTTCTAGCCGTGCTGGTGGTGCTGACTTCCTGATGCGAGTCGGTGACAAGAAGGTAACACTTAACGGAGCCACTACGACTTACCGAGAGTTGATTGGTGAGGAACAGTGGAACGCTCTCATGGTCACAGCACAACGTTCTAAGTTTGAGACTGACGCGAAGCTGAACGAGCAGTATCGCTTGGAGATTAActc                                                                                                                                                                                                                                                                                                <  1:291569/301‑1 (MQ=255)`  
             `taagagacagtcttTGAGAAGTATATCGACAACGGTCTGGTTACTGGCGCAATCCCATCTGATGCTCAAGCCACACAGCTTATAAGCCAAGCGTTCAGTGACGCTTCTAGCCGTGCTGGTGGTGCTGACTTCCTGATGCGAGTCGGTGACAAGAAGGTAACACTTAACGGAGCCACTACGACTTACCGAGAGTTGATTGGTGAGGAACAGTGGAACGCTCTCATGGTCACAGCACAACGTTCTCAGTTTGAGACTGACGCGAAGCTGAACGAGCAGTATCGCTTTGAGATTAACTCTgcgc                                                                                                                                                                                                                                                                                           <  1:245098/291‑1 (MQ=255)`  
              `ctgctgACTTCTTTGAGAAGTATATCGACAACGGTCTGGTTACTGGCGCAATCCCATCTGATGCTCAAGCCACACAGCTTATAAGCCAAGCGTTCAGTGACGCTTCTAGCCGTGCTGGTGGTGCTGACTTCCTGATGCGAGTCGGTGACAAGAAGGTAACACTTAACGGAGCCACTACGACTTACCGAGAGTTGATTGGTGAGGAACAGTGGAACGATCTCATGGTCACAGCACAACGTTCTCAGTTTGAGACTGACGCGAAGCTGAACGAGCAGTATCGCTTGGAGATTAACCCTgcgcc                                                                                                                                                                                                                                                                                          >  1:311397/1‑300 (MQ=255)`  
                  `tgACTTCTTTGAGAAGTATATCGACAACGGTCTGGTTACTGGCGCAATCCCATCTGATGCTCAAGCCACACAGCTTATAAGCCAAGCGTTCAGTGACGCTTCTAGCCGTGCTGGTGGTGCTGACTTCCTGATGCGAGTCGGTGACAAGAAGGTAACACTTAACGGAGCCACTACGACTTACCGAGAGTTGATTGGTGAGGAACAGTGGAACGCTCTCATGGTCACAGCACAACGTTCTCAGTTTGAGACTGACGCGAAGCTGAACGAGCAGTATCGCTTGGAGATTAACTCTGCGCTGAAc                                                                                                                                                                                                                                                                                      <  1:277256/301‑1 (MQ=255)`  
                       `tcttTGAGAAGTATATCGACAACGGTCTGGTTACTGGCGCAATCCCATCTGATGCTCAAGCCACACAGCTTATAAGCCAAGCGTTCAGTGACGCTTCTAGCCGTGCTGGTGGTGCTGACTTCCTGATGCGAGTCGGTGACAAGAAGGTAACACTTAACGGAGCCACTACGACTTACCGAGAGTTGATTGGTGAGGAACAGTGGAACGCTCTCATGGTCACAGCACAACGTTCTCAGTTTGAGACTGACGCGAAGCTGAACGAGCAGTATCGCTTGGAGATTAACTCTGCGCTGAACCAaga                                                                                                                                                                                                                                                                                 <  1:240456/301‑1 (MQ=255)`  
                         `aacaggaAGTATATCGACAACGGTCTGGTTACTGGCGCAATCCCATCTGATGCTCAAGCCACACAGCTTATAAGCAAAGCGTTCAGTGACGCTTCTAGCCGTGCTGGTGGTGCTGACTTCCTGATGCGAGTCGGTGACAAGAAGGTAACACTTAACGGAGCCACTACGACTTACCGAGAGTTGATTGGTGAGGAACAGTGGAACGCTCTCATGGTCACAGCACAACGTTCTCAGTTTGAGACTGACGCGAAGCTGAACGAGCAGTATCGCTTGGAGATTAACTCTGCGCTGAACCAAGAgg                                                                                                                                                                                                                                                                               <  1:135343/296‑1 (MQ=255)`  
                           `tGAGAAGTATATCGACAACGGTCTGGTTACTGGCGCAATCCCATCTGATGCTCAAGCCACACAGCTTATAAGCCAAGCGTTCAGTGACGCTTCTAGCCGTGCTGGTGGTGCTGACTTCCTGATGCGAGTCGGTGACAAGAAGGTAACACTTAACGGAGCCACTACGACTTACAGAGAGTTGATTGGTGAGGAACAGTGGAACGCTCTCATGGTCACAGCACAACGTTCTCAGTTTGAGACTGACGCGAAGCTGAACGAGCAGTATCGCTTGGAGATTAACTCTGCGCTGAACCAAGAGGAc                                                                                                                                                                                                                                                                             <  1:130239/301‑1 (MQ=255)`  
                            `gagaAGTATATCGACAACGGGCGGGGTACTGGCGCAATCCCATCTGATGCTCAAGCCACACAGCTTATAAGCCAAGCGTTCAGTGACGCTTCTAGCCGTGCTGGTGGTGCTGACTGCCTGATGCGAGTCGGTGACAAGAAGGTAACACTTAACGGAGCCACTACGACTTACCGAGAGTTGATTGGCGAGGAACAGTGGAACGCTCTCATGGTCACAGCACAACGTTCTCAGTTTGAGACTGACGCGAAGCTGAACGAGCAGTATCGCTTGGAGATTAACTCTGCGCTGAACCAAGAGGAcc                                                                                                                                                                                                                                                                            <  1:37051/301‑1 (MQ=255)`  
                              `gaAGTATATCGACAACGGTCTGGTTACTGGCGCAATCCCATCTGATGCTCAAGCCACACAGCTTATAAGCCAAGCGTTCAGTGACGCTTCTAGCCGTGCTGGTGGTGCTGACTTCCTGATGCGAGTCGGTGACAAGAAGGTAACACTTAACGGAGCCACTACGACTTACCGAGAGTTGATTGGTGAGGAACAGTGGAACGCTCTCATGGTCACAGCACAACGTTCTCAGTTTGAGACTGACGCGAAGCTGAACGAGCAGTATCGCTTGGAGATTAACTCTGCGCTGAACCAAGAGGACCCa                                                                                                                                                                                                                                                                          >  1:266067/1‑301 (MQ=255)`  
                                 `gTATATCGACAACGGTCTGGTTACTGGCGCAATCCCATCTGATGCTCAAGCCACACAGCTTATAAGCCAAGCGTTCAGTGACGCTTCTAGCCGTGCTGGTGGTGCTGACTTCCTGATGCGAGTCGGTGACAAGAAGGTAACACTTAACGGAGCCACTACGACTTACCGAGAGTTGATTGGTGAGGAACAGTGGAACGCTCTCATGGTCACAGCACAACGTTCTCAGTTTGAGACTGACGCGAAGCTGAACGAGCAGTATCGCTTGGAGATTAAGTCTGCGTTGAACAAAGAGGACCCAAgg                                                                                                                                                                                                                                                                       >  1:237998/1‑301 (MQ=255)`  
                                 `gTATATCGACAACGGTCTGGTTACTGGCGCAATCCCATCTGATGCTCAAGCCACACAGCTTATAAGCCAAGCGTTCAGTGACGCTTCTAGCCGTGCTGGTGGTGCTGACTTCCTGATGCGAGTCGGTGACAAGAAGGTAACACTTAACGGAGCCACTACGACTTACCGAGAGTTGATTGGTGAGGAACAGTGGAACGCTCTCATGGTCACAGCACAACGTTCTCAGTTTGAGACTGACGCGAAGCTGAACGAGCAGTATCGCTTGGAGATTAACTCTGCGCTGAACCAAGAGGACCCAAgg                                                                                                                                                                                                                                                                       >  1:271524/1‑301 (MQ=255)`  
                                 `gTATATCGACAACGGTCTGGTTACTGGCGCAATCCCATCTGATGCTCAAGCCACACAGCTTATAAGCCAAGCGTTCAGTGACGCTTCTAGCCGTGCTGGTGGTGCTGACTTCCTGATGCGAGTCGGTGACAAGAAGGTAACACTTAACGGAGCCACTACGACTTACCGAGAGTTGATTGGTGAGGAACAGTGGAACGCTCTCATGGTCACAGCACAACGTTCTCAGTTTGAGACTGACGCGAAGCTGAACGAGCAGTATCGCTTGGAGATTAACTCTGCGCTGAACCAAGAGGACCCAAgg                                                                                                                                                                                                                                                                       >  1:50641/1‑301 (MQ=255)`  
                                 `gTATATCGACAACGGTCTGGTTACTGGCGCAATCCCATCTGATGCTCAAGCCACACAGCTTATAAGCCAAGCGTTCAGTGACGCTTCTAGCCGTGCTGGTGGTGCTGACTTCCTGATGCGAGTCGGTGACAAGAAGGTAACACTTAACGGAGCCACTACGACTTACCGAGAGTTGATTGGTGAGGAACAGTGGAACGCTCTCATGGTCACAGCACAACGTTCTCAGTTTGAGACTGACGCGAAGCTGAACGAGCAGTATCGCTTGGAGATGAACTCTGCGCTGAACCAAGAGGACCCAAgg                                                                                                                                                                                                                                                                       >  1:288132/1‑301 (MQ=255)`  
                                 `gTATATCGACAACGGTCTGGTTACTGGCGCAATCCCATCTGATGCTCAAGCCACACAGCTTATAAGCCAAGCGTTCAGTGACGCTTCTAGCCGTGATGGTGGTGCTGACTTCCTGATGCGAGTCGGTGACAAGAAGGTAACACTTAACGGAGCCACTACGACTTACCGAGAGTTGATTGGTGAGGAACAGTGGAACGCTCTCATGGTCACAGCACAACGTTCTCAGTTTGAGACTGACGCGAAGCTGAACGAGCAGTATCGCTTGGAGATTAAcctgtctcttatacacatctcagagccc                                                                                                                                                                                                                                                                       >  1:282358/1‑274 (MQ=255)`  
                                 `gTATATCGACAACGGTCTGGTTACTGGCGCAATCCAATCTGATGCTCAAGCCACACAGCTTATAAGCCAAGCGTTCCGTGACGCTTCTAGACGTGCTGGTGGTGCTGACTTCCAAACGCGAGTCGGTGACAAAACGGTAACACTTAACGGAGACACTACGCCTTTCCGAGAGTTGATTGGTGAGTAACAGTAGAAAGGACTCATGCTCACAGCTCAATGTTCTCAGTTTGAGACTGACGCGAAGCTGAAAGAGCAGTATCGATTAGAGATTAACCATGCGCTGAACCACGAGGACCCAAgg                                                                                                                                                                                                                                                                       >  1:331214/1‑301 (MQ=255)`  
                                   `taagagACAGCGGTCTGGTTACTGGCGAAATCCCATCTGATGCTCAAGCCACACAGCTTATAAGCCAAGCGTTCAGTGACGCTTCTAGCCGTGCTGGTGGTGCTGACTTCCTGATGCGAGTCGGTGACAAGAAGGTAACACTTAACGGAGCCACTACGACTTACCGAGAGTTGATTGGTGAGGAACAGTGGAACGCTCTCATGGTCACAGCACAACGTTCTCAGTTTGAGACTGACGCGAAGCTGAACGAGCAGTATCGCTTGGAGATTAACTCTGCGCTGAACCAAGAGGACCCAAGGAc                                                                                                                                                                                                                                                                     <  1:334179/296‑1 (MQ=255)`  
                                   `atatCGACTCCGGTCTGTTTACTGGCGCACTCCCATCTGATGCTCAAGCCACACAGCTTATAAGCCAAGCGTTCAGTGACGCTTCTAGCCGTGCTGGTGGTGCTGACTTCCTGATTCGAGTCGGTGACAAGAAGGTAACACTTAACGGAGCCACTACGACTTACCGAGAGTTGATTGGTGAGGAACAGTGGAACGCTCTCATGGTCACAGCACAACGTTATCAGTTTGAGACTGACGCGAAGCTGAACGAGCAGTATCGCTTGGAGATTAACTCTGCGCTGAACCAAGAGGACCCAAGGAc                                                                                                                                                                                                                                                                     <  1:145869/301‑1 (MQ=255)`  
                                     `atCGACAACGGTCTGGTTACTGGCGCAATCCCATCTGATGCTCAAGCCACACAGCTTATAAGCCAAGCGTTCAGTGACGCTTCTAGCCGTGCTGGTGGTGCTGACTTCCTGATGCGAGTCGGTGACAAGAAGGTAACACTTAACGGAGCCACTACGACTTACCGAGAGTTGATTGGTGAGGAACAGTGGAACGCTCTCATGGTCACAGCACAACGTTCTCAGTTTGAGACTGACGCGAAGCTGAACGAGCAGTATCGCTTGGAGATTAACTCTGCGCTGAACCAagactgtctcttataca                                                                                                                                                                                                                                                                   >  1:327234/1‑287 (MQ=255)`  
                                      `tCGACACCGGCTTGTGTACTGGCGCAATCCCTTCTGATGCTCAATCCACACAGCTTATAAGCCAAGCGTTCAGTGACGCTTCTAGCCGTGCTGGTGGTGCTGACTTCCTGATGCGAGTCTGTGACAAGAAGGTAACACTTAACGGAGCCACTACGACTTACCGAGAGTTGATTGGTGAGGAACAGTGGAACGCTCTCATGGTCACAGCACAACGTTCTCAGTTTGAGACTGACGCGAAGCTGAACGAGCAGTATCGCTTGGAGATTAACTCTGCGCTGAACCAAGAGGACCAAAGGACAGc                                                                                                                                                                                                                                                                  <  1:45538/301‑1 (MQ=255)`  
                                       `cGACAACGGTCTGGTTACTGGCGCAATCCCATCTGATGCTCAAGCCACACAGCTTATAAGCCAAGCGTTCAGTGACGCTTCTAGCCGTGCTGGTGGTGCTGACTTCCTGATGCGAGTCGGTGACAAGAAGGTAACACTTAACGGAGCCACTACGACTTACCGAGAGTTGATTGGTGAGGAACAGTGGAACGCTCTCATGGTCACAGCACAACGTTCTCAGTTTGAGACTGACGCGAAGCTGAACGAGCAGTATCGCTTGGAGATTAACTCTGCGCTGAACCAAGAGGACCCAAGGAcctgt                                                                                                                                                                                                                                                                 >  1:41998/1‑297 (MQ=255)`  
                                           `aaCGGTCTGGTTACTGGCGCAATCCCATCTGATGCTCAAGCCACACAGCTTATAAGCCAAGCGTTCAGTGACGCTTCTAGCCGTGCTGGTGGTGCTGACTTCCTGATGCGAGTCGGTGACAAGAAGGTAACACTTAACGGAGCCACTACGACTTACCGAGAGTTGATTGGTGAGGAACAGTGGAACGCTCTCATGGTCACAGCACAACGTTCTCAGTTTGAGACTGACGCGAAGCTGAACGAGCAGTATCGCTTGGAGATTAACTCTGCGCTGAACCAAGAGGACCCAAGGACAGCTTggg                                                                                                                                                                                                                                                             <  1:107723/301‑1 (MQ=255)`  
                                            `aCGGTCTGGTTACTGGCGCAATCCCATCTGATGCTCAAGCCACACAGCTTATAAGCCAAGCGTTCAGTGACGCTTCTAGCCGTGCTGGTGGTGCTGACTTCCTGATGCGAGTCGGTGACAAGAAGGTAACACTTAACGGAGCCACTACGACTTACCGAGAGTTGATTGGTGAGGAACAGTGGAACGCTCTCATGGTCACAGCACAACGTTGTCAGTTTGAGACTGACGCGAAGCTGAACGAGCAGTATCGCTTGGAGATTAACTCTGCGCTGAACCAAGAGGACCCAAGGACAGCTTGGga                                                                                                                                                                                                                                                            <  1:294626/301‑1 (MQ=255)`  
                                             `cGGTCTGGTTACTGGCGCAATCCCATCTGATGCTCAAGCCACACAGCTTATAAGCCAAGCGTTCAGTGACGCTTCTAGCCGTGCTGGTGGTGCTGACTTCCTGATGCGAGTCGGTGACAAGAAGGTAACACTTAACGGAGCCACTACGACTTACCGAGAGTTGATTGGTGAGGAACAGTGGAACGCTCTCATGGTCACAGCACAACGTTCTCAGTTTGAGACTGACGCGAAGCTGAACGAGCAGTATCGCTTGGAGATTAACTCTGCGCTGAACCAAGAGGACCCAAGGACAGCTTGGgag                                                                                                                                                                                                                                                           >  1:164020/1‑301 (MQ=255)`  
                                              `ggTCTGGTTACTGGCGCAATCCCATCTGATGCTCAAGCCACACAGCTTATAAGCCAAGCGTTCAGTGACGCTTCTAGCCGTGCTGGTGGTGCTGACTTCCTGATGCGAGTCGGTGACAAGAAGGTAACACTTAACGGAGCCACTACGACTTACCGAGAGTTGATTGGTGAGGAACAGTGGAACGCTCTCATGGTCACAGCACAACGTTCTCAGTTTGAGACTGACGCGAAGCTGAACGAGCAGTATCGCTTGGAGATTAACTATGCGCTGAACCAAGAGGACCCAAGGACAGCTTGGgaga                                                                                                                                                                                                                                                          >  1:158354/1‑301 (MQ=255)`  
                                               `gTCTGGTTACTGGCGCAATCCCATCTGATGCTCAAGCCACACAGCTTATAAGCCAAGCGTTCAGTGACGCTTCTAGCCGTGCTGGTGGTGCTGACTTCCTGATGCGAGTCGGTGACAAGAAGGTAACACTTAACGGAGCCACTACGACTTACCGAGAGTTGATTGGTGAGGAACAGTGGAACGCTCTCATGGTCACAGCACAACGTTCTCAGTTTGAGACTGACGCGAAGCTGAACGAGCAGTATCGCTTGGAGATTAACTCTGCGCTGAACCAAGAGGACACAAGGACAGCTTgggctgt                                                                                                                                                                                                                                                         >  1:111008/1‑297 (MQ=255)`  
                                                        `agggCGCAATCCAGTCGGATGCTCAAGCCACACAGCTTATAAGCCAAGCGTTCAGGGACGCTTCTAGCCGTGCTGGTGGTGCTGACTTCCTGATGCGAGTCGGTGACAAGAAGGTAACACTTAAAGGAGACACTACGACTTACCGAGAGTTGATTGGTGAGGAACAGTGGAACGGTCTCATGGTCACAGCACAACGTTCTCAGTTTGAGACTGACGCGAAGCTGAACGAGCAGTATCGCTTGGAGATTAACTCTGCGCTGAACCAAGAGGATCCAAGGACAGCTTGGGAGATGCTTCAAgg                                                                                                                                                                                                                                                <  1:329088/299‑1 (MQ=255)`  
                                                         `tGGCGCAATCCCATCTGATGCTCAAGCCACACAGCTTATAAGCCAAGCGTTCAGTGACGCTTCTAGCCGTGCTGGTGGTGCTGACTTCCTGATGCGAGTCGGTGACAAGAAGGTAACACTTAACGGAGCCACTACGACTTACCGAGAGTTGATTGGTGAGGAACAGTGGAACGCTCTCATGGTCACAGCACAACGTTCTCAGTTTGAGACTGACGCGAAGCTGAACGAGCAGTATCGCTTGGAGATTAACTCTGCGCTGAACCAAGAGGACCCAAGGACAGCTTGGGAGATGCTTCAAGGt                                                                                                                                                                                                                                               <  1:150760/301‑1 (MQ=255)`  
                                                               `aaTCCCATCTGATGCTCGAGCCACACAGCTTATAAGCCAAGCGTTCAGTGACGCTTCTAGCCGTGCTGGTGGTGCTGACTTCCTGATGCGAGTCGGTGACAAGAAGGTAACACTTAACGGAGCCACTACGACTTACCGAGAGTTGATTGGTGAGGAACAGTGGAACGCTCTCATGGTCACAGCACAACGTTCTCAGTTTGAGACTGACGCGAAGCTGAACGGGCAGTATCGCTTGGAGATTAACTCTGCGCTGAACCAAGAGGACCCAAGGACAGCTTGGGAGATGCTTCAAGGTATCAAg                                                                                                                                                                                                                                         <  1:300359/301‑1 (MQ=255)`  
                                                               `aaTCCCATCTGATGCTCAAGCCACACAGCTTATAAGCCAAGCTTTCAGTGACGCTTCTATCCGTGCTGGTGGTGCTGACTTCCTGATGCGAGTCGGTGACAAGAAGGTAACACTTAACGGAGCCACTACGACTTACCGAGAGTTGATTGGTGAGGAACAGTGGAACGCTCTCATGGTCACAGCACAACGTTCTCAGTTTGAGACTGACGCGAAGCTGAACGAGCAGTATCGCTTGGAGATTAACTCTGCGCTGAACCAAGAGGACCCAAGGACAGCTTGGGAGATGCTTCAAGGTATCAAg                                                                                                                                                                                                                                         <  1:84334/301‑1 (MQ=255)`  
                                                               `aaTCCCATCTGATGCTCAAGCCACACAGCTTATAAGCCAAGCGTTCAGTGACGCTTCTAGCCGTGCTGGTGGTGCTGACTTCCTGATGCGAGTCGGTGACAAGAAGGTAACACTTAACGGAGCCACTACGACTTACCGAGAGTTGATTGGTGAGGAACAGTGGAACGCTCTCATGGTCACAGCACAACGTTCTCAGTTTGAGACTGACGCGAAGCTGAACGAGCAGTATCGCTTGGAGATTAACTCTGCGCTGAACCAAGAGGACCCAAGGACAGCTTGGgagactgtctcttatacacat                                                                                                                                                                                                                                         >  1:269612/1‑284 (MQ=255)`  
                                                                `aTCCCATCTGATGCTCAAGCCACACAGCTTATAAGCCAAGCGTTCAGTGACGCTTCTAGCCGTGCTGGTGGTGCTGACTTCCTGATGCGAGTCGGTGACAAGAAGGTAACACTTAACGGAGCCACTACGACTTACCGAGAGTTGATTGGTGAGGAACAGTGGAACGCTCTCATGGTCACAGCACAACGTTCTCAGTTTGAGACTTACGCGAAGCTGAACGAGCAGTATCGCTTGGAGATTAACTCTGCGCTGAACCAAGAGGACCCAAGGACAGCTTGGGAGATGCTTCAAGGTATCAAgg                                                                                                                                                                                                                                        >  1:178451/1‑301 (MQ=255)`  
                                                                 `tCCCATCTGATGCTCAAGCCACACAGCTTATAAGCCAAGCGTTCAGTGACGCTTCTAGCCGTGCTGGTGGTGCTGACTTCCTGATGCGAGTCGGTGACAAGAAGGTAACACTTAACGGAGCCACTACGACTTACCGAGAGTTGATTGGTGAGGAACAGTGGAACGCTCTCATGGTCACAGCACAACGTTCTCAGTTTGAGACTGACGCGAAGCTGAACGAGCAGTATCGCTTGGAGATTAACTCTGCGCTGAACCAAGAGGACCCAAGGACAGCTTGGGAGATGCTTCAAGGtctgtctct                                                                                                                                                                                                                                       >  1:22575/1‑293 (MQ=255)`  
                                                                 `tCCCATCTGATGCTCAAGCCACACAGCTTATAAGCCAAGCGTTCAGTGACGCTTCTAGCCGTGCTGGTGGTGCTGACTTCCTGATGCGAGTCGGTGACAAGAAGGTAACACTTAACGGAGCCACTACGACTTACCGAGAGTTGATTGGTGAGGAACAGTGGAACGCTATCATGGTCACAGCACAACGTTCTCAGTTTGAGACTGACGCGAAGCTGAACGAGCAGTATCGCTTGGAGATTAACTCTGCGCTGAACCAAGAGGACCCAAGGACAGCTTGGGAGATGCTTCAAGGTATCAAGGc                                                                                                                                                                                                                                       >  1:173676/1‑301 (MQ=255)`  
                                                                     `aTCTGATGCTCAAGCCACACAGCTTATAAGCCAAGCGTTCAGTGACGCTTCTAGCCGTGCTGGTGGTGCTGACTTCCTGATGCGAGTCGGTGACAAGAAGGTAACACTTAACGGAGCCACTACGACTTACCTAGAGTTGATTGGTGAGGAACAGTGGAACGCTCTCATGGTCACAGCACGACGTTCTCAGTTTGAGACTGACGCGCAGCTGAACGAGCAGTATCGCTTGGAGATTAACTCTGCGCTGAACCAAGAGGACCCACGGACAGCTTGGGAGATGCTTCCAGGTATCAAGGCTGaa                                                                                                                                                                                                                                   >  1:63307/1‑301 (MQ=255)`  
                                                                           `tGCTCAAGCCACACAGCTTATAAGCCAAGCGTTCAGTGACGCTTCTAGCCGTGCTGGTGGTGCTGACTTCCTGATGCGAGTCGGTGACAAGAAGGTAACCCTTAACGGAGCCACTACGACTTACCGAGAGTTGATTGGTGAGGAACAGTGGAACGCTCTCATGGTCACAGCACAACGTTCTCAGTTTGAGACTGTCGCGAAGCTGAACGAGCAGTATCGCTTGGAGATTAACTCTGCGCTGAACCCAGAAGACCCAAGGACAGCTTGGGCGATGCTTCAAGGTATCAAGGCTGAACTAGAt                                                                                                                                                                                                                             >  1:10421/1‑301 (MQ=255)`  
                                                                            `gCTCAAGCCACACAGCTTATAAGCCAAGCGTTCAGTGACGCTTCTAGCCGTGCTGGTGGTGCTGACTTCCTGATGCGAGTCGGTGACAAGAAGGTAACACTTAACGGAGCCACTACGACTTACCGAGAGTTGATTGGTGAGGAACAGTGGAACGCTCTCATGGTCACAGCACAACGTTCTCAGTTTGAGACTGACGCGAAGATGAACGAGCAGTATCGCTTGGAGATTAACTCTGCGCTGAACCAAGAGGACCCAAGGACAGCTTGGGAGATGCTTCAAGGTATCAAGGCTGAACTAGATa                                                                                                                                                                                                                            <  1:247633/301‑1 (MQ=255)`  
                                                                               `cAAGCCACACAGCTTATAAGCCAAGCGTTCAGTGACGCTTCTAGCCGTGCTGGTGGTGCTGACTTCCTGATGCGAGTCGGTGACAAGAAGGTAACACTTAACGGAGCCACTACGACTTACCGAGAGTTGATTGGTGAGGAACAGTGGAACGCTCTCATGGTCACAGCACAACGTTCTCAGTTTGAGACTGACGCGAAGCTGAACGAGCAGTATCGCTTGGAGATTAACTCTGCGCTGAACCAAGAGGACCCAAGGACAGCTTGGGAGATGCTTCAAGGtctgtctcttatacacatctccg                                                                                                                                                                                                                         >  1:115108/1‑279 (MQ=255)`  
                                                                               `cAAGCCACACAGCTTATAAGCCAAGCGTTCAGTGACGCATCTAGCCGTGCTGGTGGTGCTGACTTCCTGATGCGAGTCGGTGACAAGAAGGTAACACTTAACGGAGCCACTACGACTTACCGAGAGTTGATTGGTGAGGAACAGTGGAACGCTCTCATGGTCACAGCACAACGTTCTCAGTTTGAGACTGACGCGAAGCTGAACGAGCAGTATCGCTTGGAGATTAACTCTGCGCTGAACCAAGAGGACCCAAGGACAGCTTGGGAGATGCTTCCAGGTATCAAGGCTGAACTAgcagtct                                                                                                                                                                                                                         >  1:342504/1‑295 (MQ=255)`  
                                                                                 `aagagaaagagtgataAGCCAAGCGTTCAGTGGCGCTGCTAGCCGTGCTGGTGGTGCTGACTTCCTAATGCGAGTCGGTAACAAGAAGGTAACACTTAACGGAGCCACTACGACTTACCGAGAGTTGATTGGTGAGGAACAGTGGAAAGCTCTCATGGTCACAGAACAACGTTCTCAGTTTGAGACTGACGCGAAGCTGAACGAGCAGTATCGCTTGGAGAGTAACACTGCGCTGAACCAAGAGGACCAAAGGACAGCTTGGGAGATGCTTCAAGGTATCAAGGCTGAACTAGATAAGGTc                                                                                                                                                                                                                       <  1:82888/288‑1 (MQ=255)`  
                                                                                   `ccACACAGCTTATAAGCCAAGCGTTCAGTGACGCTTCTAGCCGTGCTGGTGGTGCTGACTTCCTGATGCGAGTCGGTGACAAGAAGGTAACACTTAACGGAGCCACTACGACTTACCGAGAGTTGATTGGTGAGGAACAGTGGAACGCTCTCATGGTCACAGCACAACGTTCTCAGTTTGAGACTGACGCGAAGCTGAACGAGCAGTATCGCTTGGAGATTAACTCTGCGCTGAACCAAGAGGACCCAAGGACAGCTTGGGAGATGCTTCAAggctgtctcttatacacatctacgagccc                                                                                                                                                                                                                     >  1:263805/1‑274 (MQ=255)`  
                                                                                      `tgtgtataagagacagaGCGTTCATGGACGGTTCTAGCCGTGCTGGTGGTGCTGACTTCCTGATGCGAGTCGGTGACAAGAAGGTAACACTTAACGGAGCCACTACGACTTACCGAGAGTTGATTGGTGAGGAACAGTGGAACGCTCTCATGGTCACAGCACAACGTTCTCAGTTTGAGACTGACGCGAAGCTGAACGAGCAGTATCGCTTGGAGATTAACTCTGCGCTGAACCAAGAGGACCCAAGGACAGCTTGGGAGATGCTTCAAGGTATCAAGGCTGAACTAGATAAGGTCCAAcc                                                                                                                                                                                                                  <  1:94230/285‑1 (MQ=255)`  
                                                                                      `gacaGCTTATAAGCCAAGCGTTCAGTGACGCTTCTAGCCGTGCTGGTGGTGCTGACTTCCTGATGCGAGTCGGTAACAAGAAGGTAACACTTAACGGAGCCACTACGACTTACCGAGAGTTGATTGGTGAGGAACAGTGGAACGCTCTCATGGTCACAGCACAACGTTATCAGTTTGAGACTGACGCGAAGCTGAACGAGCAGTATCGCTTGGAGATTAACTCTGCGCTGAACCAAGAGGACCCAAGGACAGCTTGGGAGATGCTTCAAGGTATCAAGGCTGAACTAGATAAGGTCCAAcc                                                                                                                                                                                                                  <  1:76265/300‑1 (MQ=255)`  
                                                                                          `gCTTATAAGCCAAGCGTTCAGTGACGCTTCTATCCGTGCTGGTGGTGCTGACTTCCTGATGCGAGTCGGTGACAAGAAGGTAACACTTAACGGAGCCACTACGACTTACCGAGAGTTGATTGGTGAGGAACAGTGGAACGCTCTCATGGTCACAGCACAACGTTCTCAGTTTGAGACTGACGCGAAGCTGAACGAGCAGTATAGCTTGGAGATTAACTCTGCGCTTAACACAGAGGACACAAGGAAAGCTTTGGTGATGATTCAAGGTATCAAGGCTGAACAAGATCAGGTCCaagcagta                                                                                                                                                                                                              >  1:127180/1‑295 (MQ=255)`  
                                                                                          `gCTTATAAGCCAAGCGTTCAGTGACGCTTCTAGCCGTGCTGGTGGTGCTGACTTCCTGATGCGAGTCGGTGACAAGAAGGTAACACTTAACGGAGCCACTACGACTTACCGAGAGTTGATTGGTGAGGAACAGTGGAACGCTCTCATGGTCACAGCACAACGTTCTCAGTTTGAGACTGACGCGAAGCTGAACGAGCAGTATCGCTTGGAGATTAACTCTGCGCTGAACCAAGAGGACCCAAGTACAGCTTGGGAGATGCTTCAAGGTATCAAGGCTGAACTAGATAAGGTCAAACCtgat                                                                                                                                                                                                              >  1:120675/1‑301 (MQ=255)`  
                                                                                          `gCTTATAAGCCAAGCGTTCAGTGACGCTTCTAGCCGTGCTGGTGGTGCTGACTTCCTGATGCGAGTCGGTGACAAGAAGGTAACACTTAACGGAGCCACTACGACTTACCGAGAGTTGATTGGTGAGGAACAGTGGAACGCTCTCATGGTCACAGCACAACGTTCTCAGTTTGAGACTGACGCGAAGCTAAACGAGCAGTATCGCTTGGAGATTAACTCTGCGCTGAACCAAGAGGACCCAAGGACAGCTTGGGAGATGCTTCAAGGTATCAAgctgtctcttatacacatctccgagccc                                                                                                                                                                                                              >  1:123257/1‑274 (MQ=255)`  
                                                                                           `cTTATAAGCCAAGCGTTCAGTGACGCTTCTAGCCGTGCTGGTGGTGCTGACTTCCTGATGCGAGTCGGTGACAAGAAGGTAACACTTAACGGAGCCACTACGACTTACCGAGAGTTGATTGGTGAGGAACAGTGGAACGCTCTCATGGTCACAGCACAACGTTCTCAGTTTGAGACTGACGTGAAGCTGAACGAGCAGTATCGCTTGGAGATTAACTCTGCGCTGAACCAAGAGGACCCAAGGACAGCTTGGGAGATGCTTCAAGGTATCAAGGCTGAACTAGATAAGGTctgtctcttat                                                                                                                                                                                                             >  1:231022/1‑291 (MQ=255)`  
                                                                                           `cTTATAAGCCAAGCGTTCAGTGACGCTTCTAGCCGTGCTGGTGGTGCTGACTTCCTGATGCGAGTCGGTGACAAGAAGGTAACACTTAACGGAGCCACTACGACTTACCGAGAGTTGATTGGTGAGGAACAGTGGAACGCTCTCATGGTCACAGCACAACGTTCTCAGTTTGAGACTGACGCGAAGCTGAACGAGCAGTATCGCTTGGAGATTAACTCTGCGCTGAACCAAGAGGACCCAAGGACAGCTTGGGAGATGCTTCAAGGTATCAAGGCTGAACTAGATAAGGTCCAACCtgatg                                                                                                                                                                                                             >  1:169777/1‑301 (MQ=255)`  
                                                                                             `tcggagatgtgtataagagacagCTTCTAGCCGTGCTGGTGGTGCTTACTTCCTGATGCGAGTCGGTGACAAGAAGGTAACACTTAACGGAGCCACTACGACTTACCGAGAGTTGATTGGTGAGGAACAGTGGAACGCTCTCATGGTCACAGCACAACGTTCTCAGTTTGAGACTGACGCGAAGCTGAACGAGCAGTATCGCTTGGAGATTAACTCTGCGCTGAACCAAGAGGACCCAAGGACAGCTTGGGAGATGCTTCAAGGTATCAAGGCTGAACTAGATAAGGTCCAACCTGATGAg                                                                                                                                                                                                           <  1:298208/279‑1 (MQ=255)`  
                                                                                                              `gTGACGCTTCTAGCCGTGCTGGTGGTGCTTACTTCCTGATGCGAGTCGGTGACACGAAGGTAACACTTCACGGTGCCACTACTACTTACCGTGAGTTGATTGGTGAGGACCAGTGGAACGCTCTCATGGTCACAGCACAACGTTCTCAGTTTGAGACTGCCGCGCAGCTGAACGAGCAGTATCGCTTGGCGATTAACTCTGCGCTGAACCAAGAGGACCCAAGGACATCTTGGGCGATTCTTCAAGGTATCAAGGCTGCACTAGATATGGTCCAACCTACTGAGCAGATGACACCACAAct                                                                                                                                                                                          >  1:40101/1‑300 (MQ=255)`  
                                                                                                              `gTGACGCTTCTAGCCGTGCTGGTGGTGCTGACTTCCTGATGCGAGTCGGTGACAAGAAGGTAACACTTAACGGAGCCACTACGACTTACCGAGAGTTGATTGGTGAGGAACAGTGGAACGCTCTCATGGTCACAGCACAACGTTCTCAGTTTGAGACTGACGCGAAGCTGAACGAGCAGTATCGCTTGGAGATTAACTCTGCGCTGAACCAAGAGGACCCAAGGACAGCTTGGGAGATGCTTCAAGGTATCAAGGCTGAACTAGATAAGGTCCAACCTGATGAGCAGATGACACCACAACg                                                                                                                                                                                          >  1:88733/1‑301 (MQ=255)`  
                                                                                                              `gTGACGCTTCTAGCCGTGCTGGTGGTGCTGACTTCCTGATGCGAGTCGGTGACAAGAAGGTAACACTTAACGGAGCCACTACGACTTACCGAGAGTTGATTGGTGAGGAACAGTGGAACGCTCTCATAGTCACAGCACAACGTTCTCAGTTTGAGACTGACGCGAAGCTGAACGAGCAGTATCGCTTGGAGATTAACTCTGCGCTGAACCAAGAGGACCCAAGGACAGCTTGGGAGATGCTTCAAGGTATCAAGGCTGAACTAGATAAGGTCCAACCTGATGAGCAGATGACACCACAACg                                                                                                                                                                                          >  1:97884/1‑301 (MQ=255)`  
                                                                                                                    `cTTCTAGCCGTGCTGGTGGTGACGACTTCCTGATGCGAGTCGGTGACAAGAAGGTAACACTTAACGGAGCCACTACGACTTACCGAGAGTGGATTGGTGAGGAACAGTGGAACGCTCTCATGGTCACAGCACAACGTTCTCAGTTTGAGACGGACGCGAAGCTGAACTAGCAGTATCGCTTGGAGATTAACTCTGCGCTGAACCAAGAGGACCCAAGGACAGCTTGGGAGATGCTTCAAGGTATCAAGGCTGAACTAGATAAGGTCCAACCTGATGAGCAGATGACACCACAACGTGAGTg                                                                                                                                                                                    <  1:1612/301‑1 (MQ=255)`  
                                                                                                                           `ccGTGCTGGTGGTGCTGACTTCCTGATGCGAGTCGGTGACAAGAAGGTAACACTTAACGGAGCCACTACGACTTACCGATAGTTGATTGGTGAGGAACAGTGGAACGCTCTCATGGTCACAGCACAACGTTCTCAGTTTGAGACTGACGCGAAGCTGAACGAGCAGTATCGCTTGGAGATTAACTCTGCGCTTAACCAAGAGGACCCAAGGACAGCTTGGGAGATGCTTCAAGGTATCAAGGCTGAACTAGATAAGGTCCAACCTGATGAGCAGATGACACCACAACGTGAGTGGCTAAtc                                                                                                                                                                             <  1:242672/301‑1 (MQ=255)`  
                                                                                                                            `cGTGCTGGTGGTGCTGACTTCCTGATGCGAGTCGGTAACAAGAAGGTAACACTTAACGGAGCCACTACGACTTACCGAGAGTTGATTGGTGAGGAACAGTTGAACGCTCTCATGGTCACAGCACAACGTTCTCAGTTTGAGACTGACGCGAAGCTGAACTAGCAGTATCGCTTGGAGATTAACTCTGCGCTGAACCAAGAGGACCCAAGGACAGCTTGGGAGATGCTTCAAGGTATCAAGGCTGAACTAGATAAGGTCAAACCTGATGAGCAGATGACACCACAACGTGAGTGGCTAAtct                                                                                                                                                                            <  1:31738/301‑1 (MQ=255)`  
                                                                                                                                  `ggtggtGCTGACTTCCTGATGCGAGTCGGTGACAAGAAGGTAGCACTTAACGGAGCCACTACGACTTACCGATAGTTGATTGGTGAGGAACAGTGGAACGCTCTCATGGTCACAGCACAACGTTCTCAGTTTGAGACTGACGCGAAGCTGAACGAGCAGTATCGCTTGGAGATTAACTCTGCGCTGAACCAAGAGGACCCAAGGACAGCTTGGGAGATGCTTCAAGGTATCAAGGCTGAACTAGATAAGGTCCAACCTGATGAGCAGATGACACCACAACGTGAGTTGCTAATCTCCGcac                                                                                                                                                                      <  1:214017/301‑1 (MQ=255)`  
                                                                                                                                            `ccTTCCCGATGCGAGTCGATGACAAGAAGGTAACACTTAACCGAGCCATTACGACTTACCGAGAGTTTATTGGTGAGGAACAGTGGAACGCTCTCATGGTCACAGCACAACGTTCTCAGTTTGAGACTGACGCGAAGCTGAACGAGCAGTATCGCTTGGAGATTAACTCTGCGCTGATCCAAGAGGACCCAAGGACAGCTTGGGAGATGCTTCAAGGTATCAAGGCTGAACTAGATAAGGTCCAACCTGATTAGCAGATGACACCACAACGTGAGTGGCTAATCTCCGCACAGGAACAAGt                                                                                                                                                            <  1:217550/300‑1 (MQ=255)`  
                                                                                                                                            `aCTTCCTGATGCGAGTCGGTGACAAGAAGGTAACACTTAACGGAGCCACTACGACTTACCGAGAGTTGATTGGTGAGGAACAGTGGAACGCTCTCATGGTCACAGCACAACGTTCTCAGTTTGAGACTGACGCGAAGCTGAACGAGCAGTATCGCTTGGAGATTAACTCTGCGATGAACCAAGAGGACCCAAGGACAGCTTGGGAGATGCTTCAAGGTATCAAGGCTGAACTAGATAAGGTCCAACCTGATGAGCAGATGACACCACAACGTGAGTGGCTAATCTCCGCACAGGAACAAGt                                                                                                                                                            <  1:117954/301‑1 (MQ=255)`  
                                                                                                                                                  `ggctcggagatgtgtataagagacagacacTGAACGGAGCCACTACGACTTACCGAGAGTTGATTGGTGAGGAACAGTTGAACGCTCTCATGGTCACAGCACAACGTTCTCAGTTTGAGACTGACGCGAAGCTGAACGAGCAGTATCGCTTGTAGATTAACTCTGCGCTGAACAAAGAGGACCCAAGGACAGCTTGGGAGATGCTTCAAGGTATCAAGGCTGAACTAGATAAGGTCCAACCTGATGAGCAGATGACACCACAACGTGAGTGGCTAATCTCCGCACAGGAACAAGTTCAGaa                                                                                                                                                      <  1:59097/275‑1 (MQ=255)`  
                                                                                                                                                   `gATGCGAGTCGGTGACAAGAAGGTAACACTTAACGGAGCCACTACGACTTACCGAGAGTTGATTGGTGAGGAACAGTGGAACGCTCTCATGGTCACAGCACAACGTTCTCAGTTTGAGACTGACGCGAAGCTGAACGAGCAGTATCGCTTGGAGATTAACTCTGCGCTGAACCAAGAGGACCCAAGGACAGCTTGGGAGATGCTTCAAGGTATCAAGGCTGAACTAGATAAGGTCCAACCTGATGAGCAGATGACACCACAACGTGAGTGGCTAATCTCCGCACAGGAACAAGTTCAGAAt                                                                                                                                                     >  1:320604/1‑301 (MQ=255)`  
                                                                                                                                                                `gACAAGAAGGTAACACTTAACGGAGCCACTACGACTTACCGAGAGTTGATTGGTGAGGAACAGTGGAACGCTCTCATGGTCACAGCACAACGTTCTCAGTTTGAGACTGACGCGAAGCTGAACGAGCAGTATCGCTTGGAGATTAACTCTGCGCTGAACCAAGAGGACCCAAGGACAGCTTGGGAGATGCTTCAAGGTATCAAGGCTGAACTAGATAAGGTCCAACCTGATGAGCAGATGACACCACAACGTGAGTGGCTAATCTCCGCACAGGAACAAGTTCAGAATCAGATGAACGCAt                                                                                                                                        >  1:73452/1‑301 (MQ=255)`  
                                                                                                                                                                       `agGTAACACTTAACGGAGCCACTACGACTTACCGAGAGTTGATTGGTGAGGAACAGTGGAACGCTCTCATGGTCACAGCACAACGTTCTCAGTTTGAGACTGACGCGAAGCTGAACGAGCAGTATCGCTTGGAGATTAACTCTGCGCTGAACCAAGAGGACCCAAGGACAGCTTGGGAGATGCTTCAAGGTATCAAGGCTGAACTAGATAAGGTCCAACCTGATGAGCAGATGACACCACAACGTGAGTGGCTAATCTCCGCACAGGAACAAGTTCAGAATCAGATGAACGCCTgtctctt                                                                                                                                 >  1:61011/1‑295 (MQ=255)`  
                                                                                                                                                                          `cagcacTTAACGGAGCCACTACGACTTACCGAGAGTTGATTGGTGAGGAACAGTGGAACGCTCTCATGGTCACAGCACAACGTTCTCAGTTTGAGACTGACGCGAAGCTGAACGAGCAGTATCGCTTGGAGATTAACTCTGCGCTGAACCAAGAGGACCCAAGGACAGCTTGGGAGATGCTTCAAGGTATCAAGGCTGAACTAGATAAGGTCCAACCTGATGAGCAGATGACACCACAACGTGAGTGGCTAATCTCCGCACAGGAACAAGTTCAGAATCAGATGAACGCATGGACGAAAGc                                                                                                                              <  1:100400/298‑1 (MQ=255)`  
                                                                                                                                                                                       `aGCCACTACGACTTACCCAGAGGTTATTGGTGAGAAACAGTGGAACGCTCTCATGGTCACAGAACAACCTTCTCAGTTTGAGACTGACCCGAAGCTGAACGAGCAGTATCGCTTGGAGATTAACTCTGCGCTGAACCAAGAGGACCCAAGGACAGCTTAGGAGATGCTTCAAGGTATCAAGGCTGAACTAGATAAGATCCAACCTGATGAGCAGATGACACCACAACGGGAGTGGCTAATCTCCGCACAGGAACAAGTTCAGAATCAGATGAACGCATGGACGAAAGCTCAGGCCAAGGct                                                                                                                 <  1:25368/301‑1 (MQ=255)`  
                                                                                                                                                                                          `cACTACGACTTACCGAGAGTTGATTGGTGAGGAACAGTGGAACGCTCTCATGGTCACAGCACAACGTTCTCAGTTTGAGACTGACGCGAAGCTGAACGAGCAGTATCGCTTGGAGATTAACTCTGCGCTGAACCAAGAGGACCCAAGGACAGCTTGGGAGATGCTTCAAGGTATCAAGGCTGAACTAGATAAGGTCCAACCTGATGAGCAGATGACACCACAACGTGAGTGGCTAATCTCCGCACAGGAACAAGTTCAGAATCAGATGAACGCATGGACGAAAGCTCAGGCCAAGGCTCTg                                                                                                              >  1:257599/1‑301 (MQ=255)`  
                                                                                                                                                                                          `cACTACGACTTACCGAGAGTTGATTGGTGAGGAACAGTGGAACGCTCTCATGGTCACAGCACAACGTTCTCAGTTTGAGACTGACGCGAAGCTGAACGAGCAGTATCGCTTGGAGATTAACTCTGCGCTGAACCAAGAGGACCCAAGGACAGCTTGGGAGATGCTTCAAGGTATCAAGGCTGAACTAGATAAGGTCCAACCTGATGAGCAGATGACACCACAACGTGAGTGGCTAATCTCCGCACAGGAACAAGTTCAGAATCAGATGAACGCATGGACGAAAGCTCAGGCCAAGGCTCTg                                                                                                              >  1:167040/1‑301 (MQ=255)`  
                                                                                                                                                                                            `cTACGACTTACCGAGAGTTGATTGGTGAGGAACAGTGGAACGCTCTCATGGTCACAGCACAACGTTCTCAGTTTGAGACTGACGCGAAGCTGAACGAGCAGTATCGCTTGGAGATTAACTCTGCGCTGAACCAAGAGGACCCAAGGACAGCTTGGGAGATGCTTCAAGGTATCAAGGCTGAACTAGATAAGGTCCAACCTGATGAGCAGATGACACCACAACGTGAGTGGCTAATCTCCGCACAGGAACAAGTTCAGAATCAGATGAACGCATGGACGAAAGCTCAGGCCAAGGcctgtct                                                                                                            >  1:285201/1‑295 (MQ=255)`  
                                                                                                                                                                                              `aCGACTTACCGAGAGTTGATTGGTGAGGAACAGTGGAACGCTCTCATGGTCACAGCACAACGTTCTCAGTTTGAGACTGACGCGAAGCTGAACGAGCAGTATCGCTTGGAGATTAACTCTGCGCTGAACCAAGAGGACCCAAGGACAGCTTGGGAGATGCTTCAAGGTATCAAGGCTGAACTAGATAAGGTCCAACCTGATGAGCAGATGACACCACAACGTGAGTGGCTAATCTCCGCACAGGAACAAGTTCAGAATCAGATGAACGCATTGACGAAAGCTCAGGctgtcccttatacac                                                                                                          >  1:279477/1‑287 (MQ=255)`  
                                                                                                                                                                                                `gACTTACCGAGAGTTGATTGGTGAGGAACAGTGGAACGCTCTCATGGTCACAGCACAACGTTCTCAGTTTGAGACTGACGCGAAGCTGAACGAGCAGTATCGCTTGGAGATTAACTCTGCGCTGAACCAAGAGGACCCAAGGACAGCTTGGGAGATGCTTCAAGGTATCAAGGCTGAACTAGATAAGGTCCAACCTGATGAGCAGATGACACCACAACGTGAGTGGCTAATCTCCGCACAGGAACAAGTTCAGAATCAGATGAACGCATGGACGAAAGCTCAGGCCAAGGCTCTGGACGAt                                                                                                        >  1:182470/1‑301 (MQ=255)`  
                                                                                                                                                                                                  `cTTACCGAGAGTTGATTGGTGAGGAACAGTGGAACGCTCTCATGGTCACAGCACAACGTTCTCAGTTTGAGACTGACGCGAAGCTGAACGAGCAGTATCGCTTGGAGATTAACTCTGCGCTGAACCAAGAGGACCCAAGGACAGCTTGGGAGATGCTTCAAGGTATCAAGGCTGAACTAGATAAGGTCCAACCTGATGAGCAGATGACACCACAACGTGAGTGGCTAATCTCCGCACAGGAACAAGTTCAGAATCAGATGAACGCATGGACGAAAGCTCAGGCCAAGGCTCTGGACGATTc                                                                                                      >  1:314671/1‑301 (MQ=255)`  
                                                                                                                                                                                                     `aCCGAGAGTTGATTGGTGAGGAACAGTGTAACGCTCTCATGGTCACAGCACAACGTTCTCAGTTTGAGACTGACGCGAAGCTGAACGAGCAGTATCGCTTGGAGATTAACTCTGCGCTGAACCAAGAGGACCCAAGGACAGCTTGGGAGATGCTTCAAGGTATCAAGGCTGAACTAGATAAGGTCCAACCTGATGAGCAGATGACACCACAACGTGAGTGGCTAATCTCCGCACAGGAACAAGTTCAGAATAAGATGAACGCATGGACGAAAGCTCAGGCCAAGGCTCTGGACGATTCCaa                                                                                                   >  1:207918/1‑300 (MQ=255)`  
                                                                                                                                                                                                                    `gTGAGGAACAGTGGAACGCTCTCATGGTCACAGCACAACGTTCTCAGTTTGAGACTGACGCGAAGCTGAACGAGCAGTATCGCTTGGAGATTAACTCTGCGCTGAACCAAGAGGACCCAAGGACAGCTTGGGAGATGCTTCAAGGTATCAAGGCTGAACTAGATAAGGTCCAACCTGATGAGCAGATGACACCACAACGTGAGTGGCTAATCTCCGCACAGGAACAAGTTCAGAATCAGATGAACGCATGGACGAAAGCTCAGGCCAAGGCTCTGGACGATTCCATGAAGTCAATGAACaa                                                                                    >  1:304688/1‑301 (MQ=255)`  
                                                                                                                                                                                                                      `gAGGAACAGTGGAACGCTCTCATGGTCACAGCACAACGTTCTCAGTTTGAGACTGACGCGAAGCTGAACGAGCAGTATCGCTTGGAGATTAACTCTGCGCTGAACCAAGAGGACCCAAGGACAGCTTGGGAGATGCTTCAAGGTATCAAGGCTGAACTAGATAAGGTCCAACCTGATGAGCAGATGACACCACAACGTGAGTGGCTAATCTCCGCACAGGAACAAGTTCAGAATCAGATGAACGCATGGACGAAAGCTCAGGCCAAGGCTCTGGACGATTCCATGAAGTCAATGAACAAAc                                                                                  >  1:7872/1‑301 (MQ=255)`  
                                                                                                                                                                                                                       `gacagcAGTGGAACGCTCTCATGGTCACAGCACAACGTTCTCAGTTTGAGACTGACGCGAAGCTGAACGAGCAGTATCGCTTGGAGATTAACTCTGCGCTGAACCAAGAGGACCCAAGGACAGCTTGGGAGATGCTTCAAGGTATCAAGGCTGAACTAGATAAGGTCCAACCTGATGAGCAGATGACACCACAACGTGAGTGGCTAATCTCCGCACAGGAACAAGTTCAGAATCAGATGAACGCATGGACGAAAGCTCAGGCCAAGGCTCTGGACGATTCCATGAAGTCAATGAACAAACt                                                                                 <  1:138842/296‑1 (MQ=255)`  
                                                                                                                                                                                                                           `aCAGTGGAACGCTCTCATGGTCACAGCACAACGTTCTCAGTTTGAGACTGACGCGAAGCTGAACGAGCAGTATCGCTTGGAGATTAACTCTGCGCTGAACCAAGAGGACCCAAGGACAGCTTGGGAGATGCTTCAAGGTATCAAGGCTGAACTAGATAAGGTCCAACCTGATGAGCAGATGACACCACAACGTGAGTGGCTAATCTCCGCACAGGAACAAGTTCAGAATCAGATGAACGCATGGACGAAAGCTCATGCCAAGGCTCTGGACGATTCCATGAAGTCAATGAACAAACtctgt                                                                             >  1:260649/1‑297 (MQ=255)`  
                                                                                                                                                                                                                                 `gAACGCTCTCATGGTCACAGCACAACGTTCTCAGTTTGAGACTGACGCGAAGCTGAACGAGCAGTATCGCTTGGAGATTAACTCTGCGCTGAACCAAGAGGACCCAAGGACAGCTTGGGAGATGCTTCAAGGTATCAAGGCTGAACTAGATAAGGTCCAACCTGATGAGCAGATGACACCACAACGTGAGTGGCTAATCTCCGCACAGGAACAAGTTCAGAATCAGATGAACGCATGGACGAAAGCTCAGGCCAAGGCTCTGGACGATTCCATGAAGTCAATGAACAAACTTGACGTAATc                                                                       <  1:251230/301‑1 (MQ=255)`  
                                                                                                                                                                                                                                        `ctcATTGTCACAGCACAACGTTCTCAGTTTGAGACTGACGCGAAGCTGAACGAGCAGTATCGCTTGGAGATTAACTCTGCGCTGAACCAAGAGGACCCAAGGACAGCTTGGGAGATGCTTCAAGGTATCAAGGCTGAACTAGATAAGGTCCAACCTGATGAGCAGATGACACCACAACGTGAGTGGCTAATCTCCGCACAGGAACAAGTTCAGAATCAGATGAACGCATGGACGAAAGCTCAGGCCAAGGCTCTGGACGATTCCATGAAGTCAATGAACAAACTTGACGTAATCGACAAGc                                                                >  1:198080/1‑301 (MQ=255)`  
                                                                                                                                                                                                                                        `ctcATGGTCACAGCACAACGTTCTCAGTTTGAGACTGACGCGAAGCTGAACGAGCAGTATCGCTTGAAGATTAACTCTGCGCTGAACCAAGAGGACCCAAGGACAGCTTGGGAGATGCTTCAAGGTATCAAGGCTGAACTAGATAAGGACCAACCTGATGAGCAGATGACAACACAACGTGAGTGGCTAATCTCCGCACAGGAACAAGTGCAGAATCAGATGAACGCATGGACGAAAGCTGAGGACAAGGCTCTGGACGATTCCATGAAGTCAATGAACAAAATTGACGTAATCGACAAgg                                                                >  1:274875/1‑300 (MQ=255)`  
                                                                                                                                                                                                                                              `gTCACGGCGCAACGTGCTCAGGTTGGGACTGACGCGAAGCTGAACGAGCAGTATCGCTTGGAGATTAACTCCGCGCTGAACCAAGAGGACCCAAGGACAGCTTGGGAGATGCTTCAAGGTATCAAGGCTGAACTAGATAATGTCCAACCTGATGCGCAGATGACCCCACAACGTGAGTGGCTAATCTCCGCACAGGAACAAGATCAGAATCAGATGAACGCATGGACGAAAGCTCAGGCCAAGGCTCTGGACGATTCCATGAAGTCAATGAACAAACTTGACGTAATCGACAAGCAATTcc                                                          <  1:55293/301‑1 (MQ=255)`  
                                                                                                                                                                                                                                              `gTCACAGCACAACGTTCTCAGTTTGAGACTGACGCGAAGCTGAACGAGCAGTATCGCTTGGAGATTAACTCTGCGCTGAACCAAGAGGACCCATGGCCAGCTTGGGAGATGCTTCAAGGTATCAAGGCTGAACTAGATAAGGTCCAACCTGATGAGCAGATGACACCACAACGTGAGTGGCTAATCTCCGCACAGGAACAAGTTCAGAATCAGATGAACGCATGGACGAAAGCTAAGGCCAAGGCTCTGGACGATTCCATGAAGTCAATGAACAAACTTGACGTAATCGACAAGCactgtc                                                          >  1:307660/1‑296 (MQ=255)`  
                                                                                                                                                                                                                                              `gTCACAGCACAACGTTCTCAGTTTGAGACTGACGCGAAGCTGAACGAGCAGTATCGCTTGGAGATTAACTCTGCGCTGAACCAAGAGGACCCAAGGACAGCTTGGGAGATGCTTCAAGGTATCAAGGCTGAACTAGATAAGGTCCAACCTGATGAGCAGATGACACCACAACGTGAGTGGCTAATCTCCGCACAGGAACAAGTTCAGAATCAGATGAACGCATGGACGAAAGCTCAGGCCAAGGCTCTGGACGATTCCATGAAGTCAATGAACAAACTTGACGTAATCGACAAGCAATTcc                                                          >  1:336582/1‑301 (MQ=255)`  
                                                                                                                                                                                                                                                   `aGCACAACGTTCTCAGTTTGAGACTGACGCGAAGCTGAACGAGCAGTATCGCTTGGAGATTAACTCTGCGCTGAACCAAGAGGACCCAAGGACAGCTTGGGAGATGCTTCAAGGTATCAAGGCTGAACTAGATAAGGTCCAACCTGATGAGCAGATGACACCACAACGTGAGTGGCTAATCTCCGCACAGGAACAAGTTCAGAATCAGATGAACGCATGGACGAAAGCTCAGGCCAAGGCTCTGGACGATTCCATGAAGTCAATGAACAAACTTGACGTAATCGACAAGCAATTCCAGAAg                                                     <  1:203255/301‑1 (MQ=255)`  
                                                                                                                                                                                                                                                         `aCGTTCTCAGTTTGAGACTGACGCGAAGCTGAACGAGCAGTATCGCTTGGAGATTAACTCTGCGCTGAACCAAGAGGACCCAAGGACAGCTTGGGAGATGCTTCAAGGTATCAAGGCTGAACTAGATAAGGTCCAACCTGATGAGCAGATGACACCACAACGTGAGTGGCTAATCTCCGCACAGGAACAAGTTCAGAATCAGATGAACGCATGGACGAAAGCTCAGGCCAAGGCTCTGGACGATTCCATGAAGTCAATGAACAAACTTGACGTAATCgctgtctcttatacacatctccga                                               >  1:18687/1‑278 (MQ=255)`  
                                                                                                                                                                                                                                                           `gTTCTCAGTTTGAGACTGACGCGAAGCTGAACGAGCAGTATCGCTTGTAGATTAACTCTGCGCTGAACCAAGAGGACCCAAGGACAGCTTGGGAGATGCTTCAAGGTATCAAGGCTGAACTAGATAAGGTCCAACCTGATGAGCAGATGACACCACAACGTGAGTGGCTAATCTCCGCACAGGAACAAGTTCAGAATCAGATGAACGCATGGATGAAAGCTCAGGCCAAGGCTCTGGACGATTCCATGAAGTCAATGAACAAACTTGACGTAATCGACAAGCAATTCCAGAAGCGAATCaa                                             >  1:200830/1‑301 (MQ=255)`  
                                                                                                                                                                                                                                                              `ctcAGTTTGAGACTGACGCGAAGCTGAACGAGCAGTATCACTTGGAGATTAACTCTGCGCTGAACCAAGAGGACCCAAGGACAGCTTGGGAGATGCTTCAAGGTATCAAGGCTGAACTAGATAAGGTCCAACCTGATGAGCAGATGACACCACAACGTGAGTGGCTAATCTCCGCACAGGAACAAGTTCAGAATCAGATGAACGCATGGACGAAAGCTCAGGCCAAGGCTCTGGACGATTCCATGAAGTCAATGAACAAACTTGACGTAATCGACAAGCAATTCCAGAAGCGAATCAACgg                                          <  1:13243/301‑1 (MQ=255)`  
                                                                                                                                                                                                                                                                      `gagaCTGACGCGAAGCTGAACGAGCAGTATCGCTTGGAGATTAACTCTGCGCTGAACCAAGAGGACCCAAGGACAGCTTGGGAGATGCTTCAAGGTATCAAGGCTGAACTAGATAAGGTCCAACCTGATGAGCAGATGACACCACAACGTGAGTGGCTAATCTCCGCACAGGAACAAGTTCAGAATCAGATGAACGCATGGACGAAAGCTCAGGCCAAGGCTCTGGACGATTCCATGAAGTCAATGAACAAACTTGACGTAATCGACAAGCAATTCCAGAAGCGAATCAACGGTGAGTggg                                  <  1:339494/301‑1 (MQ=255)`  
                                                                                                                                                                                                                                                                        `tataagagacaggCTGAACGAGCAGTATCGCTTGGAGATTAACTCTGCGCTGAACCAAGAGGACCCAAGGACAGCTTGGGAGATGCTTCAAGGTATCAAGGCTGAACTAGATAAGGTCCAACCTGATGAGCAGATGACACCACAACGTGAGTGGCTAATCTCCGCACAGGAACAAGTTCAGAATCAGATGAACGCATGGATGAAAGCTCAGGCCAAGGCTCTGGACGATTCCATGAAGTCAATGAACAAACTTGACGTAATCGACAAGCAATTCCAGAAGCGAATCAACGGTGAGTGGGtc                                <  1:233512/289‑1 (MQ=255)`  
                                                                                                                                                                                                                                                                          `aaagagacaggcggAACGAGCAGTATCGCTTGGAGATTAACTCTGCGCTGAACCAAGAGGACCCAAGGACAGCTTGGGAGATGCTTCAAGGTATAAAGGCTGAACTAGATAAGGTCCAACCTGATGAGCAGATGACACCACAACGTGAGTGGCTAATCTCCGCACAGGAACAAGTTCAGAATCAGATGAACGCATGGACGAAAGCTCAGGCCAAGGCTCTGGACGATTCCATGAAGTCAATGAACAAACTTGACGTAATCGACAAGCAATTCCAGAAGCGAATCAACGGTGAGTGGGtctc                              <  1:323632/288‑1 (MQ=255)`  
                                                                                                                                                                                                                                                                             `aCGTGAAGCTGAAGGAGCAGTATCGCTTGGAGATTAACTCTGCGCTGAACCAAGAGGACCCAAGGACAGCTTGGGAGATGCTTCAAGGTATCAAGGCTGAACTAGATAAGGTCCAACCTGATGAGCAGATGACACCACAACGTGAGTGGCTAATCTCCGCACAGGAACAAGTTCAGAATCAGATGAACGCATGGACGAAAGCTCAGGCCAAGGCTCTGGACGATTCCATGAAGTCAATGAACAAACTTGACGTAATCGACAAGCAATTCCAGAAGCGAATCAACGGTGAGTGGGtcctgtc                           >  1:228062/1‑296 (MQ=255)`  
                                                                                                                                                                                                                                                                             `aCGCGAAGCTGAACGAGCAGTATCGCTTGGAGATTAACTCTGCGCTGAACCAAGAGGACCCAAGGACAGCTTGGGAGATGCTTCAAGGTATCAAGGCTGAACTAGATAAGGTCCAACCTGATGAGCAGATGACACCACAACGTGAGTGGCTAATCTCCGCACAGGAACAAGTTCAGAATCAGATGAACGCATGGACGAAAGCTCAGGCCAAGGCTCTGGACGATTCCATGAAGTCAATGAACAAACTTGACGTAATCGACAAGCAATTCCAGAAGCGAATCAACGGTGAGTGGGTCTCAAc                           <  1:67825/301‑1 (MQ=255)`  
                                                                                                                                                                                                                                                                              `cgctAAGCTGAACGAGCAGTATCGCTTGGAGATTAACTCTGCGCTGAACCAAGAGGCCCCAAGGACAGCTTTGGAGATGCTTCAAGGTATCAAGGCTGAACTAGATAAGGTCCAACCTGATGAGCAGATGACACCACAACGTGAGTGGCTAATCTCCGCACAGGAACAAGTTCAGAATCAGATGAACGCATGGACGAAAGCTCAGGCCAAGGCTCTGGACGATTCCATGAAGTCAATGAACAAACTTGACGTAATCGACAAGCAATTCCAGAAGCGAATCAACGGTGAGTGGGTCTCAACg                          <  1:211354/301‑1 (MQ=255)`  
                                                                                                                                                                                                                                                                                       `gAACGAGCAGTATCGCTTGGAGATTAACTCTGCGCTGAACCAAGAGGACCCAAGGACAGCTTGGGAGATGCTTCAAGGTATCAAGGCTGAACTAGATAAGGTCCAACCTGATGAGCAGATGACACCACAACGTGAGTGGCTAATCTCCGCACAGGAACAAGTTCAGAATCAGATGAACGCATGGACGAAAGCTCAGGCCAAGGCTCTGGACGATTCCATGAAGTCAATGAACAAACTTGACGTAATCGACAAGCAATTCCAGAAGCGAATCAACGGTGAGTGGGTCTCAACGGATTTTAAg                 <  1:91847/301‑1 (MQ=255)`  
                                                                                                                                                                                                                                                                                        `aaCGAGCAGTATCGCTTGGAGATTAACTCTGCGCTGAACCAAGAGGACCCAAGGACAGCTTGGGAGATGCTTCAAGGTATCAAGGCTGAACTAGATAAGGTCCAACCTGATGAGCAGATGACACCACAACGTGAGTGGCTAATCTCCGCACAGGAACAAGTTCAGAATCAGATGAACGCATGGACGAAAGCTCAGGCCAAGGCTCTGGACGATTCCATGAAGTCAATGAACAAACTTGACGTAATCGACAAGCAATTCCAGAAGCGAATCAACGGTGAGTGGGTCTCAACGGATTTTAAgg                <  1:194290/301‑1 (MQ=255)`  
                                                                                                                                                                                                                                                                                           `gAGCAGTATCGCTTGGAGATTAACTCTGCGCTGAACCAAGAGGACCCAAGGACAGCTTGGGAGATGCTTCAAGGTATCAAGGCTGAACTAGATAAGGTCCAACCTGATGAGCAGATGACACCACAACGTGAGTGGCTAATCTCCGCACAGGAACAAGTTCAGAATCAGATGAACGCATGGACGAAAGCTCAGGCCAAGGCTCTGGACGATTCCATGAAGTCAATGAACAAACTTGACGTAATCGACAAGCAATTCCAGAAGCGAATCAACGGTGAGTGGGTCTCAACGGATTTTAAGGata             >  1:154841/1‑301 (MQ=255)`  
                                                                                                                                                                                                                                                                                                `gTATCGCTTGGAGATTAACTCTGCGCTGAACCAAGAGGACCCAAGGACAGCTTGGGAGATGCTTCAAGGTATCAAGGCTGAACTAGATAAGGTCCAACCTGATGAGCAGATGACCCCACAACGTGAGTGGCTAATCTCCGCACAGGAACAAGTTCAGAATCAGATGAACGCATGGACGAAAGCTCAGGCAAAGGCTCTGGACGATTCCATGAAGTCAATGAACAACCTTGACGTAATCGACAAGCAATTCCAGAAGCGAATCAACGGTGAGTGGGTCTCAACGGATTTTAAGGATATGCCa        >  1:176015/1‑301 (MQ=255)`  
                                                                                                                                                                                                                                                                                                `gTATCGCTTGGAGATTAACTCTGCGCTGAACCAAGAGGACCCAAGGACAGCTTGGGAGATGCTTCAAGGTATCAAGGCTGAACTAGATAAGGTCCAACCTGATGAGCAGATGACACCACAACGTGAGTGGCTAATCTCCGCACAGGAACAAGTTCAGAATCAGATGAACGCATGGACGAAAGCTCAGGCCAAGGCTCTGGACGATTCCATGAAGTCAATGAACAAACTTGACGTAATCGACAAGCAATTCCAGAAGCGAATCAACGGTGAGTGGGTCTCAACGGATTTTAAGGATATGCCa        >  1:47728/1‑301 (MQ=255)`  

                                                                                                                                                                                                                                                                                                          `|`                                                                                                                                                                                                                                                                                                          
`GCGTCGTCCAGACTCTGCTGACTTCTTTGAGAAGTATATCGACAACGGTCTGGTTACTGGCGCAATCCCATCTGATGCTCAAGCCACACAGCTTATAAGCCAAGCGTTCAGTGACGCTTCTAGCCGTGCTGGTGGTGCTGACTTCCTGATGCGAGTCGGTGACAAGAAGGTAACACTTAACGGAGCCACTACGACTTACCGAGAGTTGATTGGTGAGGAACAGTGGAACGCTCTCATGGTCACAGCACAACGTTCTCAGTTTGAGACTGACGCGAAGCTGAACGAGCAGTATCGCTTGAAGATTAACTCTGCGCTGAACCAAGAGGACCCAAGGACAGCTTGGGAGATGCTTCAAGGTATCAAGGCTGAACTAGATAAGGTCCAACCTGATGAGCAGATGACACCACAACGTGAGTGGCTAATCTCCGCACAGGAACAAGTTCAGAATCAGATGAACGCATGGACGAAAGCTCAGGCCAAGGCTCTGGACGATTCCATGAAGTCAATGAACAAACTTGACGTAATCGACAAGCAATTCCAGAAGCGAATCAACGGTGAGTGGGTCTCAACGGATTTTAAGGATATGCCAGTCAAC  >  NC_001604.1/28960‑29554`

  

Alignment Legend

Aligned base mismatch/match (shaded by quality score): `ATCG/ATCG` `< 3 ≤` `ATCG/ATCG` `< 11 ≤` `ATCG/ATCG` `< 24 ≤` `ATCG/ATCG` `< 36 ≤` `ATCG/ATCG` `< 38 ≤` `ATCG/ATCG`

Unaligned base: `atcg`    Masked matching base: `atcg`    Alignment gap: `‑`    Deleted base: `‑`
